# Effective time- and frequency-domain techniques for interpreting seismic precursors in groundwater level fluctuations on Jeju Island, Korea

**DOI:** 10.1038/s41598-020-64586-0

**Published:** 2020-05-12

**Authors:** Hak Soo Hwang, Se-Yeong Hamm, Jae-Yeol Cheong, Soo-Hyoung Lee, Kyoochul Ha, Cholwoo Lee, Nam-Chil Woo, Sul-Min Yun, Kwang-Hee Kim

**Affiliations:** 1 SEKOGEO Co., Ltd., Seongnam, Gyeonggi-do 13524 Republic of Korea; 2 Department of Geological Sciences, Pusan National University, Busan, 46241 Republic of Korea; 3 Korea Radioactive Waste Agency, Daejeon, 34129 Republic of Korea; 4 Groundwater Laboratory, Korea Institute of Geoscience and Mineral Resources (KIGAM), Daejeon, 34132 Republic of Korea; 5 Deep Subsurface Research Center, Korea Institute of Geoscience and Mineral Resources (KIGAM), Daejeon, 34132 Republic of Korea; 6 Department of Earth System Sciences, Yonsei University, Seoul, 03722 Republic of Korea

**Keywords:** Hydrology, Natural hazards

## Abstract

An effective method, involving time and frequency domains was developed to interpret seismic precursors by comparing groundwater-level fluctuations recorded immediately and long before the occurrence of a known earthquake. The proposed method, consisting of the pre-processing (3-point filtering, band-pass filtering, and spectrum analysis) and post-processing (weighted moving average method and histogram and spectrum analyses) stages, was applied to the groundwater-level time series measured at three monitoring wells on Jeju Island, South Korea, from 00:00 on 8 September 2016 to 00:00 on 22 September 2016. The Gyeongju earthquake (Mw 5.4) occurred at 20:32 on 12 September2016. The histogram analysis exhibited an accentuating bellshape as the total number of waveforms, including those caused by the earthquake, of the groundwater-level fluctuations increased. The weighted moving average analysis indicated that various abnormal waveforms with different periods occurred in the fluctuations approaching the occurrence of the earthquake. The periods of seismic precursors in the groundwater-level fluctuations were determined by spectrum analysis and varied among the monitoring wells. Seismic precursor responses attributable to the Gyeongju earthquake were identified at least 8 hours before the earthquake, and the method used in this study indicates its good potential to predict an impending earthquake.

## Introduction

Earthquakes are among the worst natural calamities; they typically strike without notice and cause an immediate loss of lives and damage to property. Studies on scientifically acceptable earthquake predictions began worldwide in the 1970s. Numerous seismologists and hydrogeologists have demonstrated that the stresses caused by earthquakes accompany hydrological and hydrogeological responses, such as changes in the groundwater level^1–9^ or groundwater flow^10^, changes in the chemical composition of groundwater^11–19^, increased stream discharge^3,20,21^, liquefaction of sediments^7,22,23^, and generation of new springs^7^ and mud volcanoes^7,24–26^. For instance, Barberio *et al*.^18^ interpreted groundwater flow and hydrogeochemical anomalies (As, V, and Fe) of spring water as seismic precursors of the earthquakes occurred in 2016-2017 in central Apennines.

Static stress change and dynamic stress causing earthquakes induce strain, and the strain changes fluid pressure^7,26–32^ and alters hydrogeological properties such as permeability^8,28^, which controls the rate of fluid flow. Manga and Bordsky^26^ reported that both static stress change and dynamic stress may be significant in the near field and intermediate field (i.e., within up to a few fault lengths), but only dynamic stress is larger than tidal stress in the far field (i.e., many fault lengths away). De Luca *et al*.^32^ revealed hydraulic pressure behaviour at a horizontal borehole at a distance of ~39 km from the 2016 Amatrice epicentre (Mw 6.0) in central Apennines. What is more surprising about many hydrologic responses is the large amplitude at great distances from the epicentres of earthquakes, as was the case, for example, in Fairbanks, Alaska, approximately 10,000 km from the source of the 2004 Sumatra earthquake^6^. Groundwater levels show fluctuation patterns in accordance with the compression and expansion of aquifers by dynamic stresses^34–37^. Lockner and Beeler^33^ reported with experimental observations that the fracture-pore system of intact and pre-fractured rocks can vary owing to continuous accumulated stress–strain changes within the elastic limit in those rocks before rock failures. Various coseismic mechanisms of hydrogeological responses have been proposed, such as the poroelastic response to coseismic static strain^28,38,39^, undrained consolidation of sediments^31^, clogging or unclogging of pores by oscillatory flows produced by passing seismic waves^36,40,41^, coseismic nucleation and growth of gas bubbles^42,43^, and occurrence of shaking-induced compaction or dilatation^44,45^.

Earthquake early-warning (EEW) systems have been developed to provide seconds to tens of seconds of warning before the onset of damaging ground shaking. The EEW systems, which typically use the initial portion of P-waves, are currently operational and providing warning information in Japan, Mexico, Romania, Taiwan, Turkey, and the United States: The ElarmS^46^ and Virtual Seismologist^47^ in California applied network based EEW approaches of evolutionary magnitude estimation. The RTMag^48^ approach in Southern Italy considers the predictive relationships to estimate the magnitude from peak displacement measurements. The EEW systems implemented in Japan^49^ and Mexico^50^ employ empirical (as opposed to predictive) relationships. Caprio *et al*.^51^ developed the spectral inversion method, which is based on the evolutionary computation of the displacement spectrum and direct estimation of the seismic moment and moment magnitude, for regional EEW magnitude estimations. The response spectrum method is a popular tool used for analysis and earthquake engineering for predicting earthquakes^52,53^.

Unlike most EEW systems, which are earthquake warnings triggered by the P-wave, earthquake predictions (i.e., epicentre and magnitude of an impending earthquake) have been implemented in Greece for more than three decades using the VAN method, named after P. Varotsos, K. Alexopoulos and K. Nomicos^54^. The VAN method is based on the detection of characteristic changes in the geoelectric potential, called the seismic electric signal (SES) that appears prior to earthquakes. SES detection is possible only at specific points on the Earth’s surface (sensitive points). Each sensitive point, however, can collect SESs only from a restricted number of seismic areas^55,56^. It is also challenging to distinguish the SESs from noisy geoelectric signals in highly urbanised and industrialised areas.

The frequency of earthquakes in South Korea has been increasing since instrumental recording began in 1978. Large earthquakes have occurred recently within the Yangsan fault zone in the cities of Pohang and Gyeongju. The moment magnitude (Mw) of the Pohang earthquake, which occurred on 15 November 2017, was Mw 5.4, whereas that of the Gyeongju earthquake, which occurred on 12 September 2016, was Mw 5.4 (or ML 5.8 according to the Korea Meteorological Administration). The magnitude of the Gyeongju earthquake is the largest observed in South Korea since instrumental recording commenced^57^. The Pohang earthquake occurred at a relatively shallow depth of 4.5 km and claimed more lives and destroyed more property than the Gyeongju earthquake^58^. Lee *et al*.^59^ reported that the groundwater levels had changed 2–3 min after the Gyeongju and Kumamoto earthquakes, and the amplitude of coseismic oscillations depends on the magnitude of the earthquakes. Kim *et al*.^60^ related the groundwater level anomaly and hydrogeochemical properties as well as isotopes (radon and strontium) with the 2016 Gyeongju earthquake in and around Gyeongju area. Most studies on seismic-related groundwater-level fluctuations focused on coseismic responses. Seismic precursors in high-resolution groundwater-level measurements have not been verified in actual groundwater-level data but could be expected in the continuous accumulated stress-strain changes within the elastic limit before an earthquake.

The primary purpose of this study is to develop an effective method of identifying seismic precursors in groundwater-level fluctuations caused by an impending earthquake. For the study, two partial time series consisting of fluctuations, one long before and the other immediately before the Gyeongju earthquake, were selected from the groundwater-level time series measured at the three monitoring wells. The two selected time series were compared to identify seismic precursor responses in both time and frequency domains.

### Geological and hydrogeological settings

**Figure 1:**
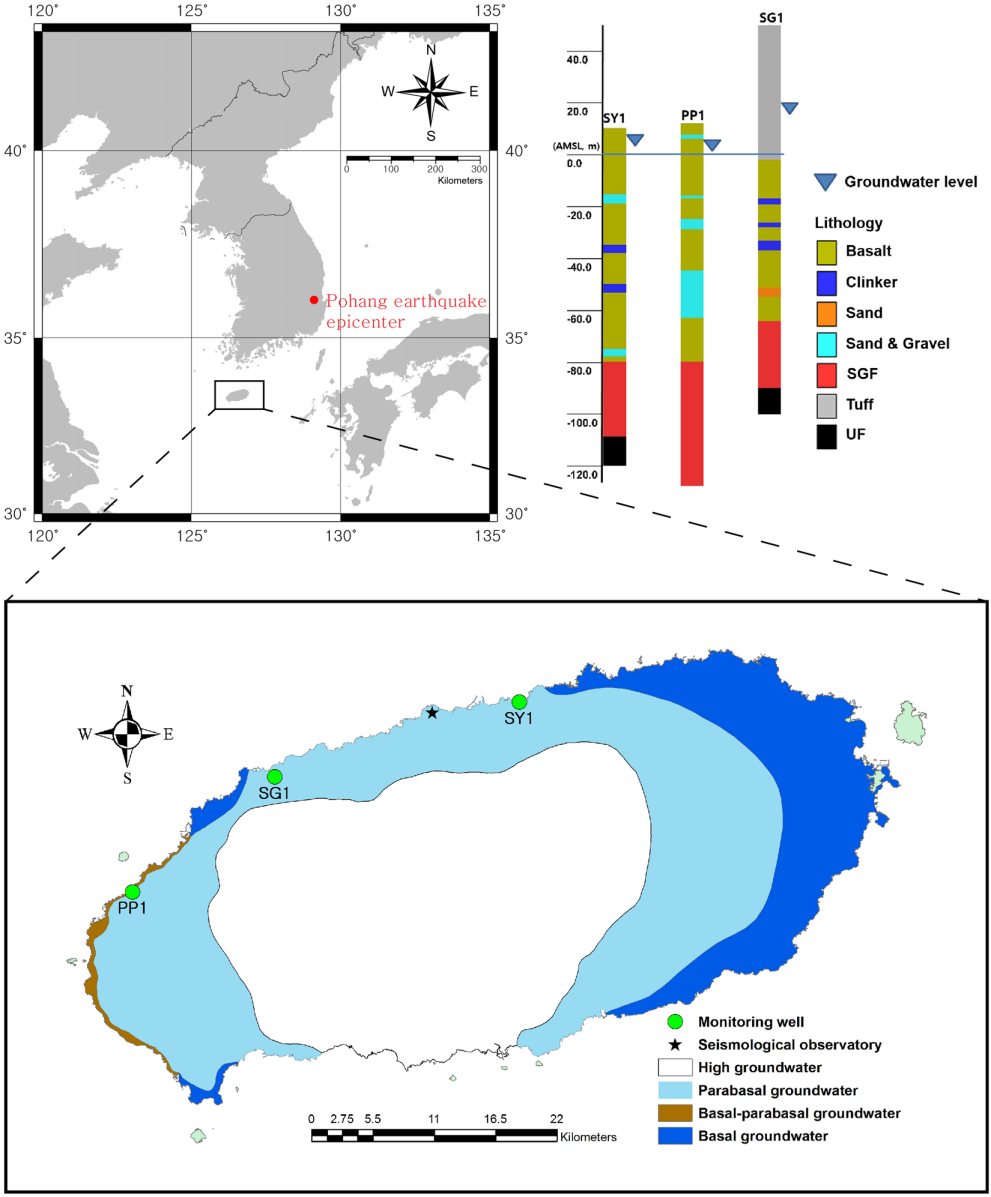
Locations of the monitoring wells for this study in relation to the high, parabasal, basal-parabasal, and basal groundwater zones on Jeju Island (lower picture) and geological columns of the monitoring wells (upper right picture).

The geology of Jeju Island typically includes volcanic rocks and sediments formed by volcanic eruptions, resulting in repeated accumulations of high- and low-permeability beds with the spatially variable hydraulic characteristics of aquifers. Clinker and sand/gravel beds, representing a permeable structure, are repeatedly intercalated between multiple layers of volcanic rock, and a mud/silt bed, with low permeability, has also been observed. According to the geological logs obtained at three monitoring wells (PP1, SG1, and SY1) in Jeju (Fig. 1), multiple layers of volcanic rocks (basalt and tuff) are distributed mostly up to approximately 65–80 m bmsl (below mean sea level), and sediment layers (unconsolidated formation: UF and Seogwipo formation: SGF) are found beneath the volcanic rock. The depth of the sedimentary layer varies according to the location.

The permeable zones below sea level of well PP1, which is located in the western part of the island and reaches 125 m bmsl, are three sand/gravel layers (3.0 m bmsl and 1.0 m thick; 22.0 m bmsl and 4.0 m thick; 42.0 m bmsl and 18.0 m thick). The permeable zones of well SG1, which is located in the north-western part of the island and reaches 100 m bmsl, are three clinker layers (18.8 m bmsl and 2.2 m thick; 26.0 m bmsl and 2.0 m thick; 33.0 m bmsl and 3.8 m thick) and one sand layer (51.2 m bmsl and 3.8 m thick). The permeable zones of well SY1, which is located in the coastal lowland area of the island and reaches119.8 m bmsl, are two sand/gravel layers (15.5 m bmsl and 3.5 m thick; 80.0 m bmsl and 3.0 m thick) and two clinker layers (35.0 m bmsl and 3.0 m thick; 50.5 m bmsl and 3.5 m thick). These permeable structures of the tuff layer, clinker, sand/gravel beds, and SGF act as groundwater flow paths and aquifers. Therefore, pressure changes within the aquifer caused by the passage of seismic waves are transmitted to these permeable structures, resulting in groundwater-level changes within the monitoring well.

The monitoring wells are located in the area of no-pumping effect. Monitoring wells SG1 and SY1 are located in the parabasal groundwater zone^61^, where fresh groundwater is not in contact with saltwater owing to an impervious or low-permeability layer at the bottom of the aquifer, whereas well PP1 is located in the basal–parabasal groundwater zone, where fresh groundwater comes into direct contact with saltwater. The groundwater levels monitored at wells PP1 and SY1 are mostly distributed approximately 3 m above mean sea level (amsl), and the groundwater level of well SG1 is located at approximately 13 m amsl. In the normal state, the groundwater levels of the monitoring wells mostly fluctuate by tidal stress, atmospheric-pressure changes, and precipitation. The peak-to-peak amplitudes of groundwater levels measured during low and high tides are approximately 30 cm and 70 cm for well PP, 20 cm and 40 cm for well SG1, and 40 cm and 80 cm for well SY1, respectively. Moreover, the transmissivity values of wells SY1 and PP1 were estimated as 4,437 m^2^/d and 52.2 m^2^/d, respectively, by an empirical equation based on a single-hole pumping test^62^.

## Methods

The data processing and analysis are performed in two stages: pre-processing and post-processing. The pre-processing stage consists of three steps: the first involves the identification of co-seismic fluctuations in the groundwater-level time series, the second involves an analysis of the time- and frequency-domain characteristics of the groundwater-level fluctuations, and the third involves the selection of two partial time series from the entire band-pass-filtered time series. In this pre-processing stage, 3-point and digital band-pass filters and spectrum analysis are used.

The post-processing stage primarily includes the identification of seismic precursor waveforms and the determination of their periods using the two partial time series produced in the pre-processing stage. The analysis in this stage is performed in both time and frequency domains. The analysis in the time domain is performed with a histogram and weighted moving average methods. The spectrum of each partial time series is produced by a fast Fourier transform (FFT), and the periods of the precursor waveforms are determined using spectrum analysis. Band-pass filters and FFTs are standard in signal processing; thus, their theoretical definitions are omitted in this paper; interested readers can refer to McClellan *et al*.^63^.

### Pre-processing stage

The consecutive difference method is typically used to identify coseismic responses that may be invisible when the responses are small in comparison with the normal differences in groundwater levels due to Earth tides, precipitation, and atmospheric-pressure variations^59,64^. However, Hwang *et al*.^64^ have demonstrated that the 3-point filter can reduce the change in groundwater levels due to tidal stresses, which is the most dominant cause of fluctuations in normal groundwater levels, more effectively than the consecutive difference method. The 3-point filter is defined as

1 $${\hat{y}}_{i}={y}_{i}-\frac{({y}_{i-l}+{y}_{i+l})}{2}(i=1+l,\cdots ,n-l),$$

where ${\hat{y}}_{i}$ and ${y}_{i}\,$are the $i$*-th* filtered groundwater level and raw groundwater level of the time series, respectively, and $N$ is the total number of data points in the time series, which must be greater than $2l$, and $L$ must be greater than 0.

### Post-processing stage

**Figure 2:**
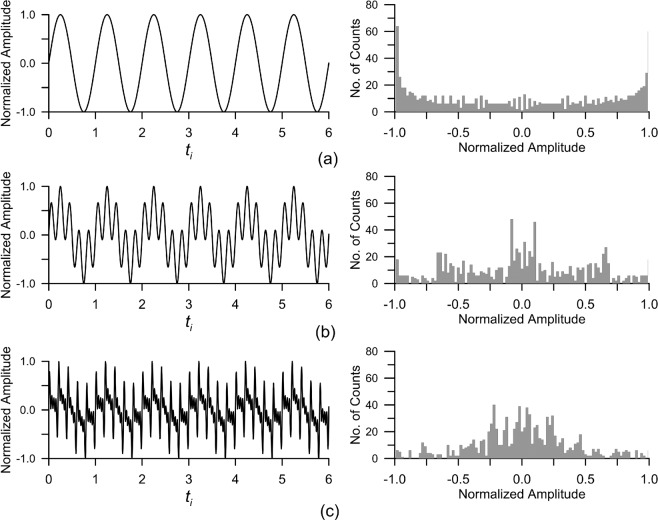
Histograms of three synthetic time series: (**a**) single waveform with a frequency of 1 Hz when $M$ = 1 in Eq. (1), (**b**) time series composed of two waveforms with frequencies of 1 and 5 Hz when $M$ = 2, (**c**) time series composed of five waveforms with frequencies of 1, 5, 10, 15, and 20 Hz when $M$ = 5. Each graph on the right corresponds to the histogram of each time series on the left.

Lockner and Beeler^65^ have reported that the fracture-pore system of intact and pre-fractured rocks can vary owing to continuous accumulated stress-strain changes within the elastic limit of the rocks before failure. These continuous accumulated stress-strain changes induce the permeability change in the rock and result in abnormal waveforms, which exhibit various periods, in normal groundwater-level fluctuations. Histogram analysis was applied to a synthetic time series to determine how the histogram changes with increase in the number of waveforms with various periods, which can be used to understand the histogram of the real groundwater levels affected by the earthquake. The synthetic time series, $A({t}_{i})$,is generated by the following equation:

2 $$A({t}_{i})=\mathop{\sum }\limits_{j=1}^{M}\,\sin (2\pi {f}_{j}{t}_{i})({t}_{i}=\frac{2\pi }{N}i,\,i=0,\cdots ,N),$$

where ${f}_{j}$ is the elementary frequency of the $j$-$th$ waveform, ${t}_{i}\,$is the $i$-*th* data point, $N\,$is the total number of data points, and $M\,$is the number of frequencies. The synthetic time series are plotted on the left side of Fig. 2 when $N$ for each time series is 980. The corresponding histogram for each time series is plotted on the right when the number of bins for each histogram is 100. As the number of waveforms with different frequencies (i.e., inverse of the period) in the time series increases, histograms approach the Gaussian distribution, which is symmetrical in the shape of a bell, around the centre. The amplitude of the waveforms that constitute the synthetic time series is selected as arbitrarily 1, since the interest in Fig. 2 is the change of histogram with the number of waveforms having different frequencies.

The weighted moving average (WMA) method is used to produce a smooth time series, which minimises the removal of waveforms with the period of interest and reduces unimportant waveforms, including white noise. The WMA value (${\hat{y}}_{j}$) at the $j$-$th$ value of the time series is defined as follows:

3 $${\hat{y}}_{j}=\frac{{\sum }_{i=j-L}^{j+L}{w}_{i}{y}_{i}}{{\sum }_{i=-{\rm{L}}}^{+L}{w}_{i}},$$

where ${y}_{i}$ is the $i$*-*$th$ value of the time series and $L$ is onehalf of the length of a moving window. The weighting coefficient (${w}_{i}$) is expressed as

4 $${w}_{i}=\frac{1}{\sqrt{2{\rm{\pi }}}}\exp \left(-\frac{\sigma }{2}{{x}_{i}}^{2}\right)\left({x}_{i}=-\,1+\frac{1}{L}i,\,i=0,\cdots ,\,2L\right),$$

where $\sigma$ is a constant that determines the envelope shape of weighting coefficients and is determined by the influence rate as

5 $$r=\frac{{\sum }_{i=L-k}^{L+k}{w}_{i}}{{\sum }_{i=-L}^{L}{w}_{i}},$$

where $k$, which is an index from the centre of the moving window, is determined by the sampling interval and the lower limit of the period band of interest. As $\sigma$ approaches zero, the WMA becomes the same as the general moving average (GMA).

### Groundwater level measurement

The groundwater level analysed in this study was measured at three wells located in Jeju Island, South Korea, for seismic monitoring (Fig. 1). Depth to water (DTW), the distance from the land surface to groundwater level, was measured by using the CTD-Diver and Baro-Diver manufactured by Eijkelkamp Co. The Baro-Diver measured the atmospheric pressure, whereas the CTD-Diver measured the pressure exerted by the water column and the atmospheric pressure in the monitoring well with a pressure resolution of 0.2 cmH_2_O. The sampling interval used to acquire the time series of groundwater levels was 1 min for the period from 00:00 on 8 September 2016 to 00:00 on 22 September 2016. The Gyeongju earthquake (Mw 5.4) occurred within this measurement period at 20:32 on 12 September 2016.

## Results and discussion

### Pre-processing results

**Figure 3:**
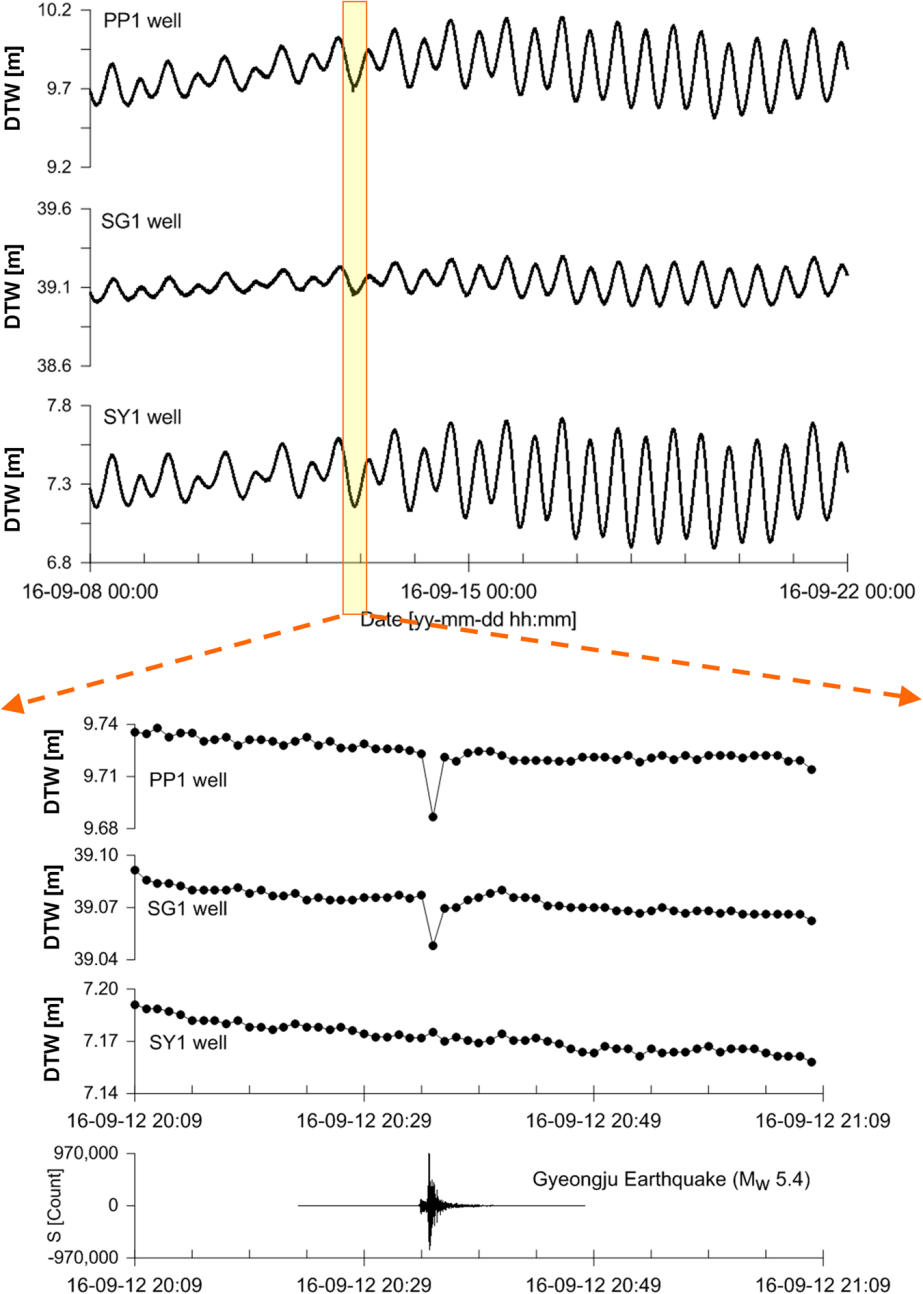
Groundwater level fluctuations at wells PP1, SG1, and SY1 measured from 00:00 on 8 September 2016 to 00:00 on 22 September 2016. The DTW stands for the depth to water. S [count] is the unit representing measured digitizing bit values of the analogue-to-digital converter (ADC) for seismic waves.

The groundwater-level time series of wells PP1, SG1, and SY1 and the seismic wave are shown in Fig. 3. The time series of the three wells are magnified in the time range from 20:09 on 12 September, 2016 to 21:08 on 12 September, 2016. The symbol (●) on the magnified time series indicates the digitised point with an interval of 1 min. The mean groundwater levels with a variance range at the three wells PP1, SG1, and SY1 were approximately 9.8 ± 0.4, 39.1 ± 0.2, and 7.3 ± 0.5 m, respectively. The dominant period of these groundwater-level fluctuations was the same as the period (~12 hours) of the semidiurnal tide that typically occurs in the western and southern seas around South Korea.

The magnified time series of PP1 and SG1 clearly exhibit the coseismic responses that are highly correlated with the seismic wave, whereas the time series of SY1 does not exhibit any coseismic responses, which can be attributed to two possible reasons. First, a sampling interval of 1 min may be too long to reveal the coseismic responses. Second, the natural frequency of well SY1, which depends on the hydrological and geological conditions of the well, may not match the frequency of the stress-strain changes due to the earthquake^66^. The primary reason is considered to be the former rather than the latter (i.e., rather than an association with the natural frequency) because the three magnified time series show that only one digitising point is present on the recorded coseismic waveforms. The lower period limit of the seismic wave is generally known to be approximately 1 second. The period of the coseismic waveforms can be estimated to be larger than the lower period limit of the seismic wave of approximately 1 s, but to obtain reasonable coseismic waveforms, considering the Nyquist frequency, the sampling rate must be at least 2 Hz.

**Figure 4:**
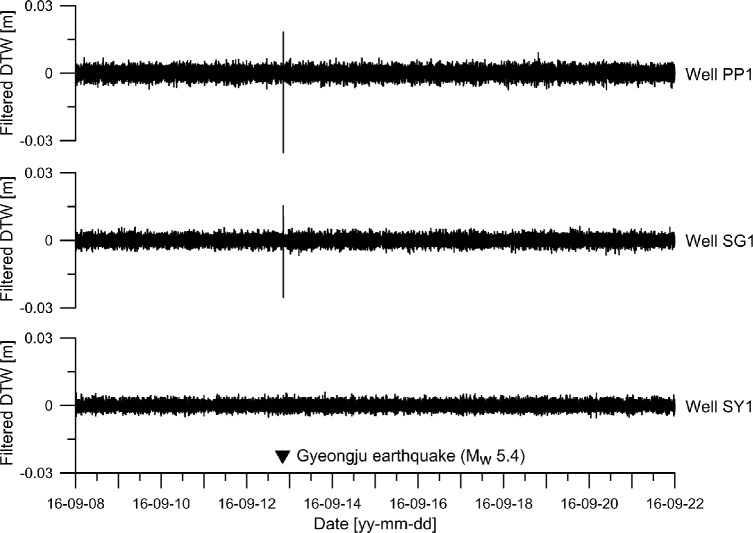
Three-point-filtered time series produced by applying the 3-point filter to the raw groundwater level fluctuations at wells PP1, SG1, and SY1.

The filtered time series produced by the 3-point filter with $l$ = 1 in Eq. (1) are presented in Fig. 4. Under the reasonable assumption that the groundwater-level changes due to atmospheric pressure variations, Earth tides, and precipitation are linear during a 2-min interval ($l\,$ = 1), the 3-point filtered time series of each well was adjusted for the average groundwater level, which is constant, as well as the groundwater-level changes due to atmospheric-pressure variations, Earth tides, and precipitation. The spikes in the 3-point filtered time series of wells PP1 and SG1 are highly correlated with the occurrence of the earthquake (▼). As the raw time series of well SY1 shown in Fig. 3, despite the efficiency of the 3-point filter that can identify coseismic responses that may be invisible in the case of coseismic responses that are small compared to the normal fluctuations of groundwater levels, the 3-point filtered time series of well SY1 does not exhibit any spikes associated with the earthquake because of the improper sampling interval as mentioned earlier.

**Figure 5:**
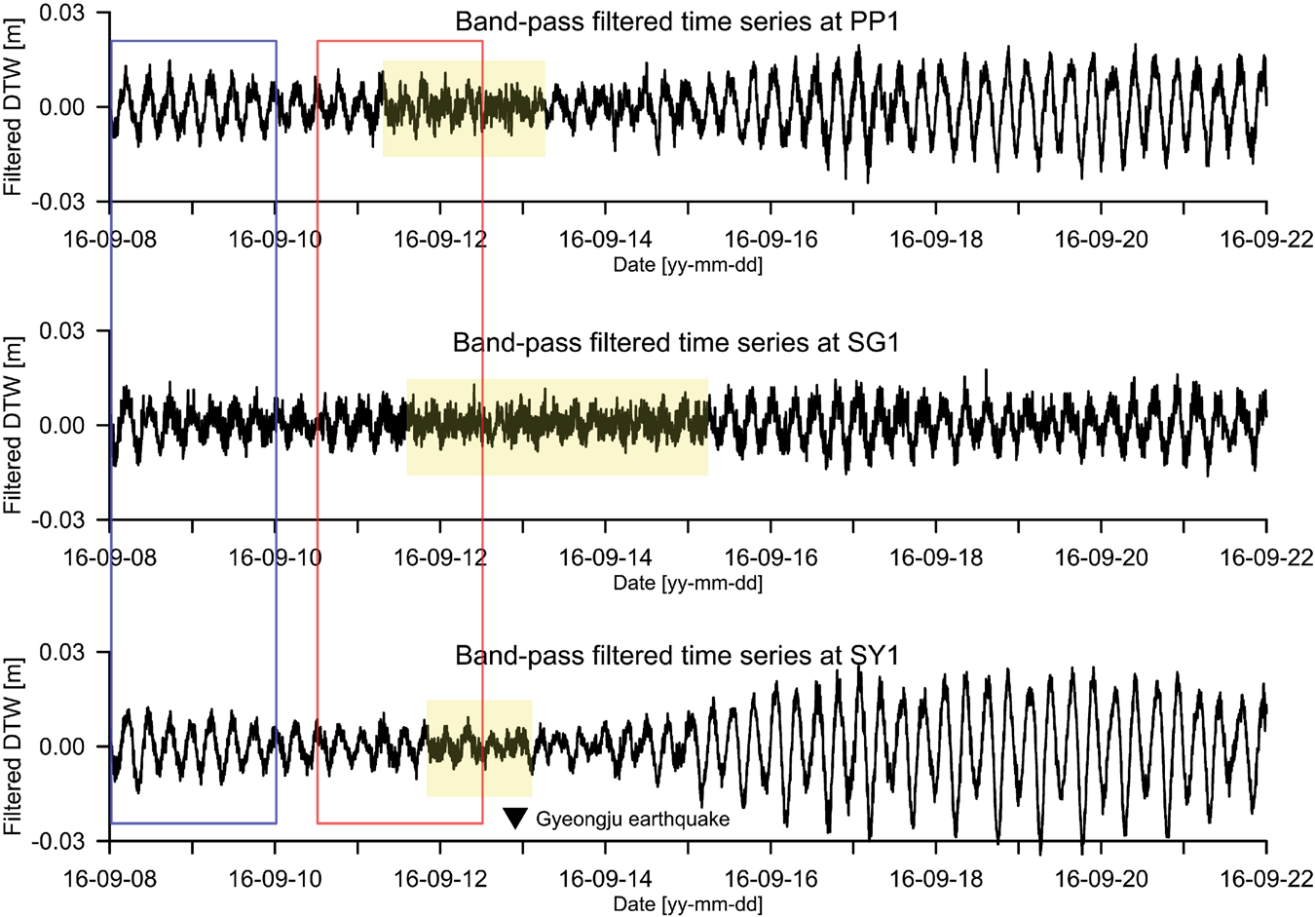
Band-pass filtered time series obtained by applying the band-pass filter to the raw time series at wells PP1, SG1, and SY1.

To understand the fluctuations of the groundwater levels within an interested period range of ~ 10 to ~400 min^43^, a band pass filter with a band-pass periodic range of 8 to 420 minutes was applied to the raw fluctuations. The upper, middle, and lower graphs shown in Fig. 5 correspond to the band-pass filtered time series for wells PP1, SG1, and SY1, respectively. Each band-pass-filtered time series shows that the filtered amplitude is between −0.03 and +0.03 m. The band-pass filtered time series of groundwater fluctuations were corrected for atmospheric pressure variations, diurnal and semidiurnal tides, and the mean groundwater level. The dominant waveform in the band-pass filtered time series is repeated approximately four times a day (~6 hours). The filtered fluctuations in the yellow-filled box show that the dominant waveform is contaminated by abnormal waveforms with much shorter periods than ~6 hours.

Two partial time series with a duration of 2 days were selected from the band-pass filtered time series at each well. The first selected time series (blue box) ranges from 00:00 on 8 September 2016 to 00:00 on 10 September 2016, whereas the second selected time series (red box) ranges from 12:00 on 10 September 2016 to 12:00 on 12 September 2016. The two series ended 4,112 and 512 min, respectively, before the occurrence of the Gyeongju earthquake. Therefore, compared with the first selected time series, the second can be assumed to have been relatively more affected by the earthquake.

**Figure 6:**
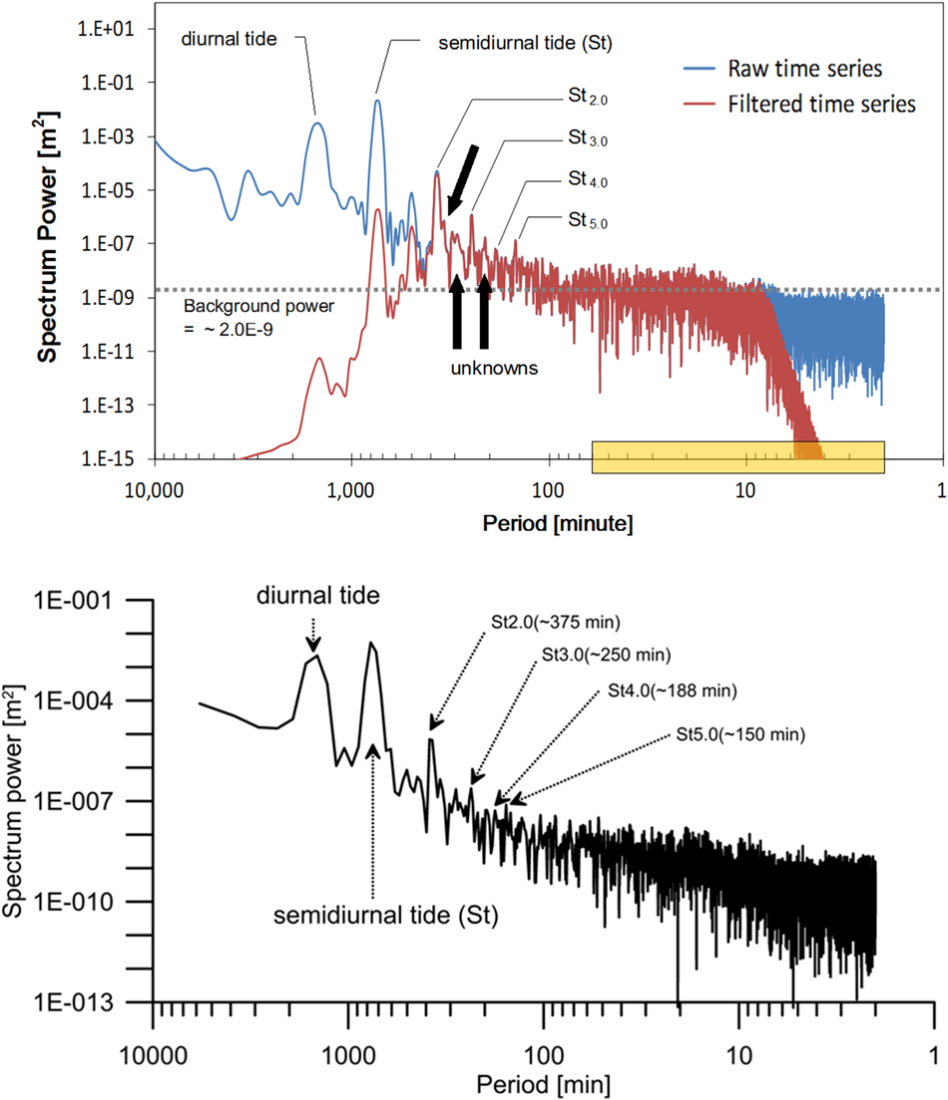
Power spectra of the raw and band-pass filtered time series at well SY1.

The power spectra produced from the raw (blue) and band-pass filtered (red) time series of well SY1 are presented on the top of Fig. 6. The background spectral power of 2.0E-09 m^2^ for well SY1 was defined by a maximum spectral power at periods less than 8 min, whereas those of PP1 and SG1 were 4.0E-09 m^2^. The most dominant spectral power was concentrated at periods of approximately 750 min (semidiurnal: St), followed by a period of 1,500 min (diurnal tide) and the St harmonics. The periods of the first (St_2.0_) and second (St_4.0_) even harmonics were 375 and 188 min, respectively, whereas the periods of the first (St_3.0_) and second (St_5.0_) odd harmonics were 250 and 150 min, respectively. White noise, which typically occurs at all frequencies less than the Nyquist frequency in any time series, is evident at periods less than 60 min (yellow box on the period axis) compared with other periods. The power spectrum shown on the bottom of Fig. 6 was produced from the groundwater-level fluctuations measured at well SY1 from 00:00 on 3 October 2015 to 00:00 on 11 October 2015. No earthquakes had occurred in South Korea, Japan, or China during the measurement period or more than 10 days before or after the measurement period. Based on the earthquake-free power spectrum, the unknowns with periods of 334, 304, 287, and 208 min (black arrows in the top diagram) are interpreted to reflect normal groundwater-level fluctuations and not the earthquake. The spectrum of the band-pass filtered time series (red) indicates that the spectral powers at the periods excluding the band-pass periodic range are remarkably reduced without any additional changes within the band-pass periodic range^67^.

**Figure 7:**
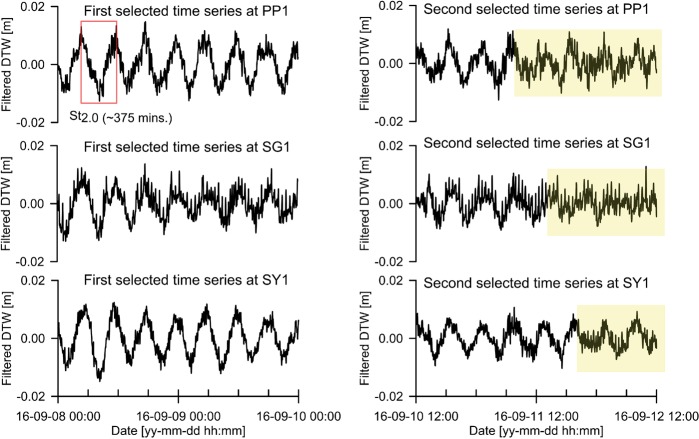
First and second selected time series at wells PP1, SG1, and SY1. The first selected time series at each well is on the left. The second selected time series at each well is on the right.

Two partial time series (Fig. 7) were selected from each of the entire band-pass filtered time series presented in Fig. 5. The first time series is used as background groundwater-level fluctuations to identify abnormal waveforms included in the second selected time. The period of dominant waveforms (red box) in the first and second selected time series at each well was at 375 min (St_2.0_).The first selected time series of well SG1 was relatively noisier than those of wells PP1 and SY1. The yellow-filled portionon the second time series of each well clearly shows that the dominant waveforms were broken by abnormal waveforms with different periods shorter than St_2.0_.

### Post-processing results

**Figure 8:**
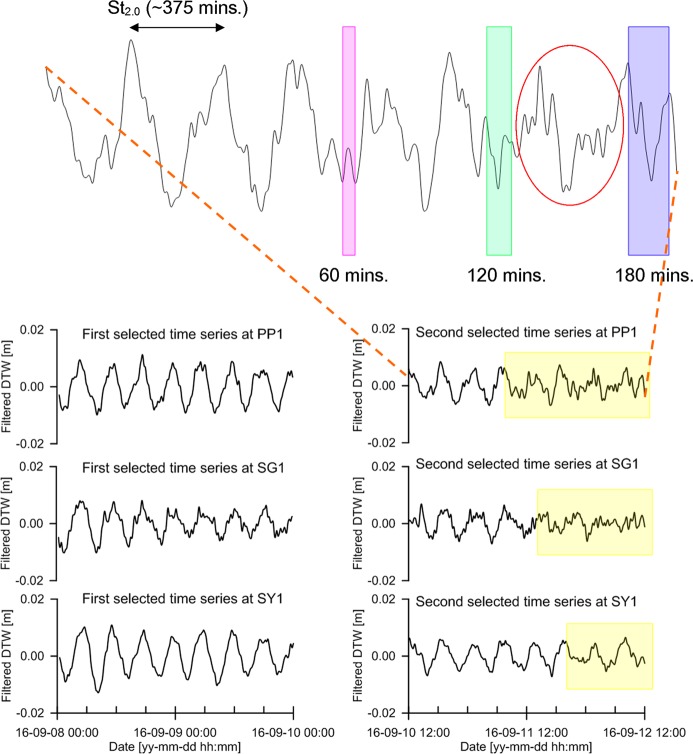
First and second time series obtained by applying the WMA to the first and second selected time series, when $L$ = $30$ and σ = 13.

Applications of the WMA method to the first and second selected time series, when *L* = 30 and σ = 13, are presented in Fig. 8. The graph in the upper part is magnified from the weighted moving averaged time series of the second selected time series at well PP1. All the time series in the lower part correspond to the WMA of the first and second selected time series at each well. The value of *L* was determined as 30 to reduce the influence of white noise at periods less than 60 min. With the given length of the moving window (2 *L* = 60 min), the value of σ was determined as 13 when the influence rate ($r$) in Eq. (5) was 48%. The WMA loses the functionality to reduce white noise at periods less than 60 min when $r$, in the range of ±4 min from the centre of the moving window, is too high. Conversely, when $r$ is too low, the WMA loses the functionality to minimise the removal of waveforms with periods in the range of 8–60 min. The waveform with a period of St_2.0_ is prominent in both the first and second selected time series obtained by the WMA. The abnormal waveforms with various periods, which are not present in the first selected time series, clearly appear in the yellow-coloured box of the second selected time series at each well. To confirm these abnormal waveforms, a portion of the series was magnified, with the pink, green, and purple windows corresponding to the waveform periods of 60, 120, and 180 min, respectively. The period of the waveform under the pink-coloured window is a little more than 60 min. The period of the waveform under the green-coloured window is approximately little less than 120 min. The period of the waveform under the purple-coloured window is approximately 180 min. The waveforms with various periods less than 60 min appear in the red ellipse.

**Figure 9:**
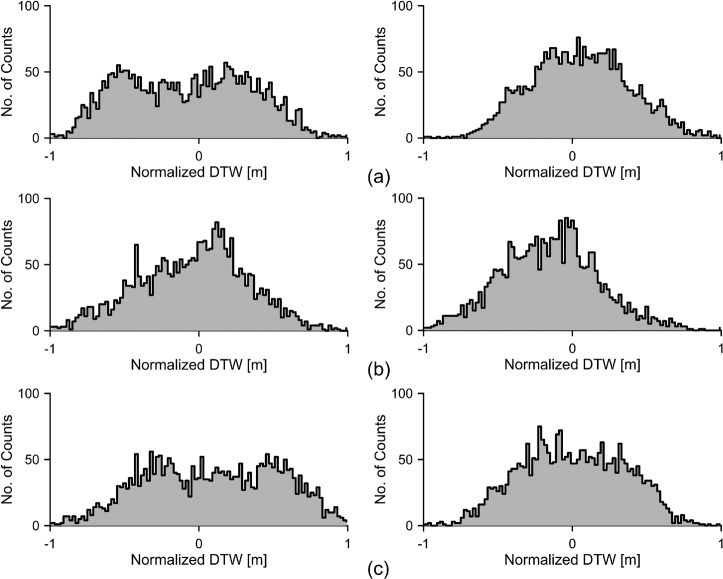
Histograms of (**a**) well PP1, (**b**) well SG1, and (**c**) well SY1. The histogram of the first selected time series is on the left. The histogram of the second selected time series is on the right.

**Figure 10:**
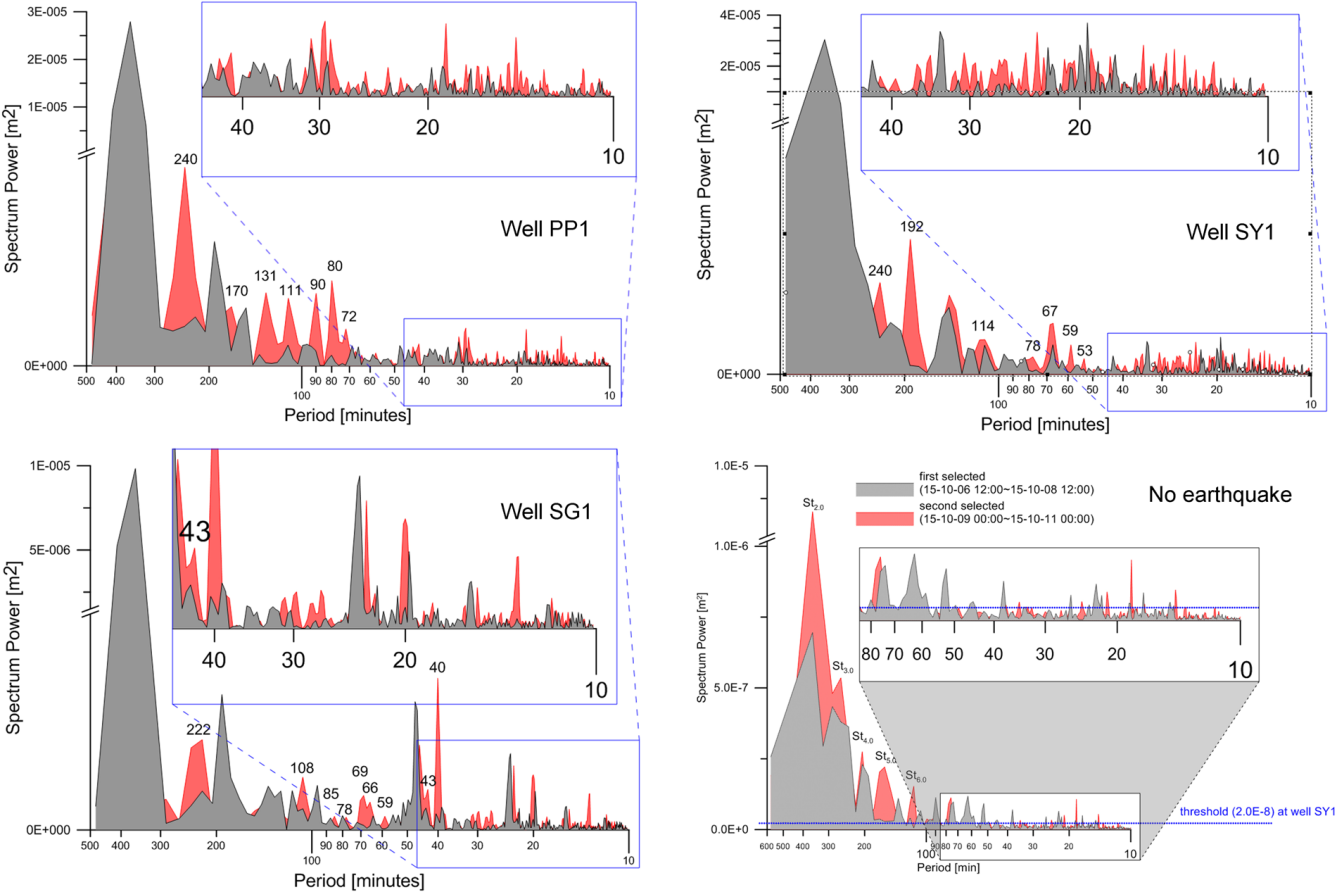
Power spectra of the first and second selected time series at wells PP1, SG1, and SY1. The grey-filled section is the spectrum of the first selected time series. The red-filled area is the spectrum of the second selected time series.

Histograms of the first and second selected time series, when the number of bins in each histogram is 100, are shown in Fig. 9. Compared with the histograms of the first selected time series at wells PP1 and SY1, each histogram of the second selected time series exhibits a more bell-shaped form that is nearly symmetric about the centre. However, both histograms for well SG1 exhibit almost identical bell-shaped forms. According to the histogram analysis of the synthetic time series presented in Fig. 2, this difference between well SG1 and wells PP1 and SY1 could be understood by counting the number of waveforms that constitute the time series. The number of waveforms was counted with the number of periods at which spectral powers are greater than a threshold. The threshold for each well was determined as 10 times the background spectral power, considering the spectral power of white noise. The numbers of periods at which the spectral powers were greater than 4.0E-08 m^2^ were 23 and 39 in the first and second selected time series at well PP1, respectively. At well SY1, the numbers of periods at which the spectral powers were greater than 2.0E-08 m^2^were 23 and 37 in the first and second selected time series, respectively. The number of periods at which the spectral powers were greater than a threshold of 4.0E-08 m^2^was 30 in each of the first and second selected time series at well SG1. According to the analysis of two partial time series selected from earthquake-free groundwater level fluctuations at well SY1 (referred to bottom graph in Fig. 10), the number of periods in which the spectral powers exceeded the threshold were 19 and 17, respectively, in the first and second selected time series. Therefore, although more case studies are required, all the selected time series of wells PP1, SG1, and SY1 presented in Fig. 7 can be considered to be differentially contaminated by the earthquake, and the fluctuations of groundwater levels at well SG1 exhibits relatively more and earlier earthquake effects than the other two wells. If a general background histogram about normal fluctuations of groundwater levels is prepared, the histogram and correlation analyses can be effectively used to make an initial diagnosis for an impending earthquake in real time. For the proper initial diagnosis, the general histogram at a monitoring well should be created and continuously updated using groundwater level fluctuations, which do not contain noises such as earthquakes and local pumping activities, over time.

The periods of abnormal waveforms were determined by comparing the power spectrum of the second selected time series with that of the first selected time series (Fig. 10). The three graphs at the top correspond to the power spectra of wells PP1, SG1, and SY1, and the graph on the bottom shows those of two partial time series which were selected from the fluctuations of groundwater levels in periods without earthquakes. The grey-filled area is the power spectrum of the first selected time series, whereas the red-filled area is that of the second time series. Four criteria to determine the periods of abnormal waveforms were considered. First, the periods of diurnal tides, St, St_2.0_, St_3.0_, St_4.0_, St_5.0_, and the four unknowns that were identified by the power spectrum of groundwater levels in periods without earthquakes should be excluded. Second, the spectral power of the second selected time series should exist in a period in which that of the first selected times series does not exist. Third, the spectral power in a specific period must be greater than the threshold defined for the histogram analysis. Fourth, though the spectral power of the first selected time series at a specific period exists, that of the second selected time series at the specific period should be clearly reinforced. The identified periods of the abnormal waveforms are presented in Table 1. The maximum period (240 min) of the abnormal waveform occurs in wells PP1 and SY1, whereas the minimum period (11.2 min) of the abnormal waveform occurs in well PP1. The periods of 43 and 67 min at which the spectral powers of the waveforms are reinforced are also found at wells SG1 and SY1, respectively. The period difference of the seismic precursor responses between the monitoring wells can be referred to research papers that describe that seismic precursor responses depend on the natural frequency of a well^41^ and the tensor-sensitivity of borehole–aquifer systems^7,8,68,69^. The well that contained the largest number (31) of periods of abnormal waveforms is SY1, followed by well PP1 (number of periods: 26) and well SG1 (number of periods: 21). The ratios of the number of abnormal waveforms to the total number of waveforms included in the second selected time series were 76, 70, and 84% at wells PP1, SG1, and SY1, respectively.

**Table 1:** Periods of abnormal waveforms identified at wells PP1, SG1, and SY1.

Wells	Periods ≥ 50 minutes	Counts	Periods <50 minutes	Counts
PP1	240.0, 169.5, 131.0, 110.8, 90. 0, 80.0, 72.0	7	43.7, 41.8, 39.0, 31.8, 31.0, 29.2, 28.8, 25.2, 23.2, 18.8, 17.3, 16.7, 16.0, 15.2, 14.8, 14.4, 14.1, 13.0, 11.2	19
SG1	221.6, 107.6, 84.5, 77.8, 68.5, 65.5, 58.7	7	44.7, 43.0, 40.0, 31.0, 29.7, 27.2, 23.0, 22.3, 20.0, 18.2, 16.0, 15.3, 13.3, 11.8	14
SY1	240.0, 192.0, 114.0, 77.8, 67.0, 58.8, 53.4	7	36.4, 34.7, 31.7, 30.6, 29.4, 28.8, 27.7, 26.9, 26.4, 25.7, 24.4, 23.4, 22.9, 21.2, 20.4, 19.2, 17.8, 15.8, 15.2, 14.6, 14.1, 13.6, 13.0, 12.2,	24

According to the following considerations, the abnormal waveforms identified at wells PP1, SG1, and SY1 are interpreted as the seismic precursor responses caused by the Gyeongju earthquake. First, even if the wells are more than 30 km apart, the three monitoring wells exhibit a high correlation in the appearance time of the abnormal waveforms. Second, during measurements, there is not any hydraulic event that causes this high correlation between the wells, other than the earthquake. Third, the spectra of two partial time series selected from the fluctuations of groundwater levels during an earthquake-free period (bottom graph in Fig. 10) are similar to each other except for the spectral powers of the semidiurnal harmonics and show clear differences from the spectrum of groundwater levels before the earthquake.

## Conclusions

It is very difficult to identify the difference between two power spectra during earthquake and earthquake-free periods. Therefore, in order to extract seismic precursors, in this study, both time- and frequency-domain techniques were applied to the groundwater level fluctuations of two selected time series.Two partial time series consisting of groundwater fluctuations at the three monitoring wells, one long before and the other immediately before the Gyeongju earthquake, were selected to identify seismic precursor responses in both time and frequency domains.

The dominant periods of the groundwater-level fluctuations measured at three monitoring wells on Jeju Island, South Korea, have been confirmed as approximately 1,500 min (diurnal tide), 750 min (semidiurnal tide: St), the harmonics of St, and a few periods of unknown events. The periods of the first and second even harmonics were 375 min (St_2.0_) and 250 min (St_3.0_), respectively, whereas those of the first and second odd harmonics were 188 min (St_4.0_) and 150 min (St_5.0_), respectively. The few unknowns with periods of approximately 334, 304, 287, and 208 min have been interpreted as not caused by the Gyeongju earthquake but reflect the normal groundwater-level fluctuations at the monitoring well. Based on these confirmed periods, the comparison analysis of the two partial groundwater-level fluctuations (one relatively far before the earthquake and the other immediately before the earthquake) shows that the ratio of abnormal waveforms caused by the earthquake to the total number of waveforms comprising partial groundwater-level fluctuations close to the occurrence of the earthquake exceeds 70%, and the periods of abnormal waveforms, which range from approximately 240–11.2 min, were confirmed to be different between the monitoring wells. Hence, the abnormal waveforms are interpreted as seismic precursors attributable to the Gyeongju earthquake, and these seismic precursors have been identified to have occurred at least 8 hours before the earthquake.

From the result of this study, we recognize the necessity of additional monitoring wells with accumulated groundwater level data for detecting precursors of earthquakes. Hence, the future research will be focused to develop sophisticated hardware and software systems in order to finally achieve the goal of predicting impending earthquakes with more clearly understanding hydrogeological behaviour of aquifers and/or fractured zones in bedrock. The developed system will be equipped with adaptive processing server for updating the background spectrum of normal groundwater levels.The proper measurement system for groundwater level precursor may possess a bit-resolution of ~0.03 mm (a dynamic range of 2 m, 16-bit ADC (analogue to digital convertor), a selective gain of 1, and low-pass filter with a high-cut frequency of 1/600. Once above mentioned systems will be built, the method proposed in this study can be potentially to be utilized to predict coming earthquakes.
